# Factors Affecting Radiation Protection Behaviors among Emergency Room Nurses

**DOI:** 10.3390/ijerph18126238

**Published:** 2021-06-09

**Authors:** Sookkyoung Park, Yaki Yang

**Affiliations:** 1 Department of Nursing, College of Nursing, Jeonbuk National University, Jeonju 54896, Korea; yoursky@jbnu.ac.kr; 2 Department of Nursing, Wonkwang University, Iksan 54538, Korea

**Keywords:** emergency nursing, radiation protection, environment, knowledge, behavior

## Abstract

This study aimed to examine factors affecting radiation protection behaviors among emergency room nurses by assessing knowledge about radiation protection and attitude towards radiation protection, employing a cross-sectional design. Subjects were a convenience sample of 129 nurses working in the emergency rooms of three advanced general hospitals. Data were collected using self-report questionnaires and analyzed using *t*-test, ANOVA, Pearson correlation coefficients, and multiple regression. There were significant relations between knowledge about radiation protection and attitude towards radiation protection (r = 0.34, *p* < 0.001), knowledge about radiation protection and radiation protection behaviors (r = 0.37, *p* < 0.001), and attitude towards radiation protection and radiation protection behaviors (r = 0.33, *p* < 0.001). The factors affecting radiation protection behaviors were radiation protection knowledge (β = 0.12, *p* = 0.045), attitude towards radiation protection (β = 0.17, *p* = 0.009), the experience of radiation protection education (β = 0.27, *p* < 0.001), and wearing of protective equipment (β = 0.29, *p* < 0.001). The governments, hospital administrators, and radiation protection agencies should strengthen their radiation defense environment to protect emergency room nurses from radiation. Research and development of radiation defense equipment and the medical examination of emergency room nurses should be carried out, radiation defense behavior protocols should be developed, radiation defense education opportunities should be provided, and the use of defense equipment should be encouraged.

## 1. Introduction

In modern medicine, techniques using radiation are constantly developing, and are being used in various fields, from diagnosis to treatment [1]. The number of radiation workers at medical institutions was 89,025 persons in 2018, representing a 25.2% increase since 2014; the number of nurses increased 1.7-fold to 8374 persons in Korea, which was the largest increase compared to other occupations, and the number of radiation workers is forecast to continue increasing in the future [2]. Due to the increased use of radiation in medicine, the risk of exposure to radiation during nursing work will inevitably increase, which has made the safety and control of radiation an important issue.

The annual effective dose limit in medicine is ≤50 mSv, and the 5-year cumulative dose limit is ≤100 mSv, while the annual mean exposure of nurses is 0.22 mSv in Korea [3]. Although medical radiation is classified as low-dose, there are no standards regarding the lowest harmful dose in humans, and so even a low dose can have negative effects on the body [4]. There are two sides to radiation; while it can be useful when handled appropriately, negligent or careless handling of radiation can cause negative effects of exposure for not only the handler, but also patients and caregivers [5]. Depending on the level of exposure, it can even cause severe impairment, including cataracts, skin erythema, hair loss, infertility, cancer, and genetic disease [4]. For this reason, it is crucial to pay attention to interventions to minimize health problems due to exposure during radiation work.

The International Commission on Radiological Protection (ICRP) stipulates that the annual exposure level to irradiation of radioactive rays be below 20 mSv, up to 50 mSv for a year, and up to a cumulative 100 mSv for five years [6]. In addition, the commission recommends the education and procedure of safety work relevant to radioactive rays, securing and using pertinent protective equipment, and provision of means to minimize exposure to irradiation of radioactive rays including effective monitoring programs to secure safety for employees working in the area of radioactive rays [7]. Complying with the rules on the safety management of diagnostic radiation generators in the medical law will significantly decrease the risk of exposure of medical personnel to irradiation with radioactive rays. Mandatory education on the safety of radioactive rays (more than 6 h per year for employees working in the area of radioactive rays) does not apply to nurses not classified as employees working in the area of radioactive rays. Only the senior person in charge of safety management in each institution is required to take the education once a year, and they particularly limited to a certain domain; thereby, regular continuing educational courses are absent for the safety management of nurses [8].

In the emergency room (ER), mobile X-ray machines are essential for the diagnosis and treatment of patients who cannot be moved to the X-ray room, severe trauma patients, and urgent patients. These devices are not only used in small- to medium-sized hospitals, and are used to scan over 100 patients per day at most large hospitals [9]. When performing mobile X-ray imaging in the ER or intensive care unit (ICU), a radiation shielding screen must be used for protection; however, these screens are difficult to drag around while imaging due to their size and weight, and they are inconvenient to use, which inevitably leads to increased radiation exposure [10]. In a study that measured exposure in ER workers during mobile X-ray imaging, the mean radiation exposure was 0.50 mSv; the mean exposure per hour of work was highest for emergency medical technicians (EMTs, 2.03 mSv), followed by nurses (0.83 mSv) and emergency medicine doctors (0.60 mSv) [11].

As medical personnel who spend a long time next to patients and play an important role in evaluating and observing the patient’s condition, nurses are at high risk of radiation exposure during working hours [12]. It is difficult to achieve proper shielding for ER nurses during mobile X-ray imaging because the nurse often has to provide support by holding the patient in position or preventing life support devices from detaching [13], and nurses have been found to be at the highest risk of radiation exposure during certain intervals [14].

Radiation protection behaviors are behaviors that protect the body from radiation exposure [5]. Examples of these behaviors include wearing protective equipment or standing far away from the radiation source, but the variety and quantity of protective equipment required by nurses are lacking in clinical settings [10,15].

In order to practice the appropriate behaviors, it is first essential to foster positive attitudes and accurate knowledge of these behaviors [16]. Insofar as knowledge and attitude are useful factors in understanding and changing human behavior, they can be included among the major factors for behavioral change. Therefore, in order to reinforce radiation protection behaviors, there is a need for knowledge and positive attitudes regarding radiation protection [17]. The main factors affecting radiation protection behaviors by ER nurses can be divided into personal factors and environmental factors [5,15,16]. The major personal factors have been identified as knowledge about radiation protection and attitude towards radiation protection [18], and the main environmental factor has been identified as the radiation protection environment [19]. The radiation protection environment can be further divided into facilities, radioprotective equipment, and the administrative environment for promoting radiation protection behaviors [5], but since this is a problem that requires administrative support from medical institutions, it is difficult to achieve significant changes in a short time. For this reason, in order to investigate factors affecting the radiation protection behaviors of ER nurses, the present study focused on the personal factors of knowledge about radiation protection and attitude towards radiation protection.

Lack of knowledge and awareness about radiation exposure among radiation workers increases the risk of radiation exposure [20]. In a previous study on the radiation protection knowledge needed to reduce the risk of exposure, ER nurses showed less knowledge than operating room (OR) and ICU nurses, who have a higher frequency of radiation exposure [21]. Nurses with more radiation protection knowledge have been reported to display a higher level of radiation protection behaviors, highlighting the necessity of education to increase radiation protection knowledge [19,21]. Attitude towards radiation protection represents the level of awareness of efforts to reduce the risk of radiation exposure, and more positive attitudes are associated with a higher likelihood of performing radiation protection behaviors to prevent exposure [5]. Thus, attitude towards radiation protection is extremely important for performing radiation protection behaviors.

Studies on radiation protection behaviors have mostly focused on radiographers. In terms of studies on nurses, there have mainly been studies investigating the relationships of radiation protection behaviors with knowledge about radiation protection knowledge and attitude towards radiation protection among nurses working in places with a large number of radiation-emitting appliances, such as the OR, endoscopy room, or ICU. The ER is a place that requires rapid, accurate diagnosis, and in this environment patients’ conditions can change suddenly. Mobile X-ray machines are used frequently in the ER since they make it possible to rapidly identify changes in a patient’s condition in an open space. However, emergency room nurses are not classified as radiation-related workers. It is necessary to determine the degree of radiation defense behavior of emergency room nurses, but such research is insufficient [22].

This study investigated the effects of knowledge about radiation protection and attitude towards radiation protection on the radiation protection behaviors of ER nurses in order to provide basic data to support the development of strategies to increase radiation protection behaviors among ER nurses. The objective of this study was to measure knowledge about radiation protection, attitude towards radiation protection, and radiation protection behaviors among ER nurses, and to identify the factors affecting radiation protection behaviors.

## 2. Methods

### 2.1. Study Design

This was a descriptive study to measure knowledge about radiation protection, attitude towards radiation protection, and radiation protection behaviors among ER nurses, and to identify the factors affecting radiation protection behaviors.

### 2.2. Participants

The participants in this study were a convenience sample of nurses working in the ER of one of seven advanced general hospitals in G City and J Province, South Korea. Using G*Power 3.1.9.4 with a significance level (α) of 0.05, power of 0.95, effect size of 0.15, and 14 predictive variables, the minimum required sample size was calculated to be 194 persons. Those with less than 3 months of experience working in the emergency room were excluded. Accounting for dropouts, 220 questionnaires were distributed and 205 responses were received. After excluding incomplete or insincere responses, 198 responses were included in the final analysis.

### 2.3. Measurement

#### 2.3.1. Knowledge about Radiation Protection

Knowledge about radiation protection was measured using an instrument originally developed by Han et al. [5] for use on radiation workers at medical institutions and modified by Kim et al. [19]. The instrument consists of 16 questions in total, including questions on the relationship between the dose of exposure and irradiated area, the wearing of thermoluminescent dosimeters, the purpose of lead aprons and duration of use, the radiosensitivity of tissues and the relationship with genetics, outcomes of radiation exposure during pregnancy, the purpose of using protective equipment, radiation shields, types of legally designated personal dosimeter, conditions for radiation work during pregnancy, awareness of legally designated education, annual exposure limits, theoretical knowledge related to general radiation protection behaviors, health examinations for radiation workers, and the relationship between radiation intensity and distance from the source. Each question is responded to with “True,” “False,” or “Not sure”; correct answers are scored 1 point, and incorrect answers and “Not sure” are scored 0 points. The range of possible scores is 0–16 points, and higher scores indicate better knowledge of radiation protection. At the time of development, the reliability of the instrument was shown by Cronbach’s α = 0.69, and in the present study, Kuder–Richardson formula 20 (KR-20) = 0.61.

#### 2.3.2. Attitude towards Radiation Protection

Attitude towards radiation protection was measured using an instrument originally developed by Han et al. [5] and modified by Lee [21]. The instrument consists of a total of 10 questions scored on a 5-point Likert scale from “Strongly disagree” (1 point) to “Strongly agree” (5 points). The range of possible scores is 10–50 points, and higher scores indicate a more positive attitude towards reducing the risk of radiation exposure and more likelihood of performing radiation protection behaviors. The instrument showed Cronbach’s α = 0.93 at the time of development and Cronbach’s α = 0.90 in the present study.

#### 2.3.3. Radiation Protection Behaviors

Radiation protection behaviors were measured using an instrument originally developed for radiation workers at medical institutions by Han et al. [5] and modified by Kang and Lee [8]. The instrument consists of a total of 18 questions, including questions on wearing personal dosimeters, whether they make efforts to reduce radiation exposure, wearing protective equipment, adjusting radiation distance and time, the management and inspection of protective equipment, radiation cautions for pregnant women, health examinations, regular education, use of a shield, shielding facilities, response in the event of failure to wear protective equipment, radiation protection by administrators, discussion with radiographers for radiation protection, and alerts when using radiation. Each question is scored on a 5-point Likert scale from “Never [perform this behavior]” (1 point) to “Always.” The range of possible scores is 18–90 points, with higher scores indicating a greater performance of radiation protection behaviors. The instrument’s reliability was shown by Cronbach’s α = 0.85 at the time of development and Cronbach’s α = 0.95 in the present study.

### 2.4. Ethical Consideration

Before commencing the study, approval was obtained from the W University Hospital institutional review board (WKIRB-201912-SB-092). The study was conducted between 16 February and 16 April 2020 at 7 advanced general hospitals located in G City and J Province. The investigators directly explained the objectives of the study and the method of completing the questionnaire to the participants; those participants who voluntarily consented to participate in the study provided their signature and completed the questionnaire. Participants were informed that they could refuse to participate in the study or withdraw at any time and they would not suffer any disadvantages. Participants were informed in advance that a gift in return would be provided after the survey was completed because prepaid incentives are believed to increase the likelihood of survey response [23]. After completing the questionnaire, participants were provided with a small wallet. The responses were collected in opaque envelopes to avoid leaking personal information, and participants were informed that the responses would be destroyed after 3 years of storage. The time required to complete the questionnaire was approximately 10–15 min.

### 2.5. Data Analysis

SPSS/WIN 26.0 was used to analyze the collected data. The frequency, percentage, mean, and standard deviation were obtained for participants’ general characteristics, knowledge about radiation protection, attitude towards radiation protection, and radiation protection behaviors. Differences in participants’ radiation protection behaviors were analyzed using independent *t*-tests and one-way ANOVA, and Scheffé’s test was used for post-hoc analysis. The relationships between knowledge about radiation protection, attitude towards radiation protection, and radiation protection behaviors were analyzed using Pearson’s correlation coefficient. The factors affecting radiation protection behaviors were analyzed using multiple regression.

## 3. Results

### 3.1. General Characteristics of Participants

The majority of the participants were female (188 persons, 94.9%), and the most common age group was 20–29 years (85 persons, 42.9%). Most of the participants had no spouse (108 persons, 54.5%), and the most common education status was university graduate, bachelor’s degree (135 persons, 68.2%). The most common total career group was more than 10 years (77 persons, 38.9%), and the most common response for career in present unit was less than 2 years (73 persons, 36.9%). The most common response for subjective health status was “average” (125 persons, 63.1%). There were 108 persons (54.5%) who reported no experience of radiation protection education, 190 persons (96.0%) who reported that radiation protection education was necessary, and 177 persons (89.4%) who reported that they intended to participate in radiation protection education. There were 124 persons (62.6%) who reported wearing protective equipment and 102 persons (51.5%) who reported that they had received radiation-related health examinations (Table 1).

### 3.2. Knowledge about Radiation Protection, Attitude towards Radiation Protection, and Radiation Protection Behaviors

The participants’ mean score for knowledge about radiation protection was 10.56 (±2.34) out of 16 points. The participants’ mean score for attitude towards radiation protection was 4.35 (±0.53) out of 5 points. The participants’ mean score for radiation protection behaviors was 3.45 (±0.81) (Table 2).

### 3.3. Differences in Radiation Protection Behaviors According to the General Characteristic

Radiation protection behaviors showed statistically significant relationships with experience of radiation protection education (*t* = 6.32, *p* < 0.001), necessity of radiation protection education (*t* = 1.05, *p* = 0.024), wearing of protective equipment (*t* = 6.56, *p* < 0.001), and experience of radiation-related health examinations (*t* = 3.60, *p* < 0.001) (Table 1).

### 3.4. Correlations among Knowledge about Radiation Protection, Attitude towards Radiation Protection, and Radiation Protection Behaviors

The correlations were analyzed between knowledge about radiation protection, attitude towards radiation protection, and radiation protection behaviors. Knowledge about radiation protection and attitude towards radiation protection showed a significant positive correlation (r = 0.34, *p* < 0.001). Knowledge about radiation protection and radiation protection behaviors showed a significant positive correlation (r = 0.37, *p* < 0.001). Attitude towards radiation protection and radiation protection behaviors showed a significant positive correlation (r = 0.33, *p* < 0.001) (Table 3).

### 3.5. Factors Affecting Radiation Protection Behaviors

Stepwise multiple regression was used to identify the main factors affecting the radiation protection behaviors of ER nurses. The variables used in the regression equation were knowledge about radiation protection and attitude towards radiation protection, which showed significant correlations with radiation protection behaviors, as well as the general characteristics that were associated with significant differences in radiation protection behaviors, which were as follows: experience of radiation protection education, willingness to participate in radiation protection education, wearing of radioprotective equipment, and experience of radiation-related health examinations. Categorical variables were converted to dummy variables, and the autocorrelation of the dependent variables and the multicollinearity were tested. The Durbin–Watson statistic was 1.926, indicating that there was no autocorrelation of the dependent variable. The correlations between the independent variables were in the range −0.36 to 0.22; since there were no correlations ≥0.80, the explanatory variables were shown to be independent. The tolerance was ≥0.1 (0.730–0.962), and the variance inflation factor (VIF) was <10 (1.039–1.334), demonstrating the absence of multicollinearity. Therefore, the data in this study were determined to be suitable for regression analysis. The regression model was significant (F = 15.99, *p* < 0.001), and the adjusted coefficient of determination (Adj. R^2^), which is a measure of the model’s explanatory power, was 0.313. The factors with significant effects on radiation protection behaviors were radiation protection knowledge (β = 0.12, *p* = 0.045), attitude towards radiation protection (β = 0.17, *p* = 0.009), the experience of radiation protection education (β = 0.27, *p* < 0.001), and wearing of protective equipment (β = 0.29, *p* < 0.001), which had a combined explanatory power of 31.3%. The strongest predictor of radiation protection behaviors was wearing of protective equipment (β = 0.29, *p* < 0.001) (Table 4).

## 4. Discussion

This study explored the relationships between knowledge about radiation protection, attitude towards radiation protection, and radiation protection behaviors among ER nurses, and analyzed the factors affecting radiation protection behaviors. In this way, the objective of the study was to prepare basic data for supporting the development of educational programs to protect ER nurses from radiation exposure and strengthen the radiation protection behaviors of ER nurses. The major implications of the study results are discussed below.

### 4.1. Interpretation of Results

The main factors affecting radiation protection behaviors, in descending order, were wearing protective equipment, experience of radiation protection education, attitude towards radiation protection, and knowledge about radiation protection. The total explanatory power of these factors for radiation protection behaviors was 31.3%. The factor with the strongest effect on radiation protection behaviors was wearing protective equipment. This finding is supported by previous studies, where the main factor affecting radiation protection behaviors was reported to be the presence or absence of protective equipment in one study of ER nurses [22], and the suitability of radiation protection equipment in a study of OR nurses [24].

During radiologic tests, it is important to logically lower the dose of exposure as much as possible through the appropriate adjustment of the three main principles of radiation protection: time, distance, and shielding [25]. In this regard, wearing radioprotective equipment can be considered an essential measure to reduce radiation exposure in radiation workers. Radioprotective facilities and equipment include the ceiling, floor, and walls of the X-ray imaging room, the shielding walls of mobile medical vehicles equipped with radiation-emitting devices, and the protective shield used with mobile X-ray machines. When diagnostic radiation-emitting devices are used in medical institutions, it is imperative to set up a radiation area to reduce exposure [26], but when mobile X-ray machines are used in the ER, it is often impractical to use the radiation shield due to its large size and weight. Since the ER contains many severe and critical patients, there is a high volume of work that needs to be resolved at once, and priorities can change depending on the circumstances, frequently leading to occasions when the required radioprotective gear cannot be worn [21]. Radiation protection guidelines for interventional radiological procedures advise that radiation workers should wear the appropriate radioprotective gear, including lead aprons, lead glasses, and thyroid shields, since secondary radiation scattered from patients or walls can cause negative effects on the eyes, hands, and thyroid gland [27]. However, safety and control regulations for diagnostic radiation-emitting devices only indicate lead aprons as essential radioprotective equipment. Radiation directed towards the patient can be categorized as primary radiation, which generates the medical image; and secondary radiation, which is scattered to the surroundings and is unrelated to the medical image. Secondary radiation is a cause of exposure in people standing nearby and should be shielded as far as possible [26]. As such, legislation regarding essential radioprotective equipment needs to be expanded beyond only lead aprons, and more protective equipment needs to be made available to encourage its use. To strengthen radiation protection behaviors by ER nurses, radioprotective equipment and facilities need to be made available, and organizational efforts are needed to promote their use. To this end, hospitals and related companies should invest in research and development to improve the performance of radiation protection facilities with equipment that is portable and convenient to use. This will also require support at the governmental level.

Experience of radiation protection education was also found to be a major factor affecting radiation protection behaviors. In a study on the effects of radiation safety and control education using leaflets for ICU nurses [28], education improved radiation safety and control behaviors by enhancing awareness of radiation safety and control and self-care from radiation exposure. Another study investigating the effects of educational videos on radiation safety and control [29] also found that education was effective at improving radiation protection behaviors. To improve radiation protection behaviors, based on these findings, it will be important to provide education not only for nurses, but for all radiation workers. Among the participants in the present study, 45.5% of nurses reported experience of radiation protection education, while 96% of nurses reported that radiation protection education was required, demonstrating that radiation safety and control education for ER nurses needs to be expanded to provide more opportunities to acquire knowledge about radiation protection.

In the present study, attitude towards radiation protection was identified as a major factor influencing radiation protection behaviors, which was consistent with a study by Han and Kwon [30]. The participants’ mean attitude score was 4.35 out of 5 points, which was similar to a previous study of ER nurses [22]. Given that a positive attitude towards radiation protection can enhance radiation protection behaviors [12], nurses’ efforts to protect themselves need to be prioritized in order to reduce the gap between nurses’ attitudes towards radiation protection and their actual radiation protection behaviors. In the present study, attitude towards radiation protection showed a stronger influence on radiation protection behaviors than knowledge about radiation protection, which is consistent with a previous study on radiographers [30]. On the other hand, one study on endoscopy nurses reported that knowledge about radiation protection did not have a significant effect on radiation protection behaviors, which differs from the results of the present study. One might consider repeating the studies across different hospital sizes and target populations. It will also be necessary to explore the specific individual effects of knowledge about radiation protection and attitude towards radiation protection on radiation protection behaviors.

The participants in this study showed a score of 3.45 (±0.81) out of 5 points for radiation protection behaviors. Among individual questions, the highest score was for “Care should be taken to avoid exposure to radiation when the worker is pregnant,” and the lowest score was for “Personal dosimeters should be worn.” This is similar to the results of a study on OR nurses that used the same instrument [8]. Wearing personal dosimeters when performing radiation-related work makes it possible for individuals to measure their own exposure and estimate the extent of radiation harm. In order to distribute personal dosimeters for each individual and make their use mandatory, department managers should check whether workers are wearing personal dosimeters before starting work, or workers should be encouraged to check for themselves using a self-report checklist.

When the correlations between knowledge about radiation protection, attitude towards radiation protection, and radiation protection behaviors were analyzed, there was a statistically significant positive correlation between knowledge about radiation protection and radiation protection behaviors, which was consistent with previous studies [31]. This shows that more knowledge about radiation protection was associated with better performance of radiation protection behaviors; thus, in order to improve radiation protection behaviors by ER nurses, it is important for the nurses to acquire thorough knowledge about radiation protection. There was also a significant positive correlation between attitude towards radiation protection and radiation protection behaviors, which was similar to previous studies [10,18,22]. In the present study, there was a significant positive correlation between knowledge about radiation protection and attitude towards radiation protection. This differs slightly from another study on nurses, where there was a weak but non-significant positive correlation between these variables [32], and so a replication study will need to be performed on a wider range of participants.

The participants in this study were not receiving radiation-exposure-related health testing, with only 48.5% reporting experience of radiation-related health examinations. Although specialized health testing is not mandatory for ER nurses, given that proper protective facilities are not available for many ER nurses who are continually exposed to low-dose radiation, even if there are no immediate physical effects, there is a risk of stochastic effects in relation to low doses. Hence, there is a need to establish legal criteria for radiation-exposure health testing for ER nurses.

The International Commission on Radiological Protection (ICRP) recommends that organizations make their own rules and protocols for radiation defense and that members are familiar with and adhere to them. Safety guidelines for medical radiation through the rules on the safety management of diagnostic radiation generators in the medical law in Korea include management, supervision, assessment, and testing of radiologic equipment; identification, provision, and appropriate use of personal protective equipment; stimulating the development of protective equipment from manufacturers; utilizing mobile shields; and maintaining distance from the source [33]. Nurses show more radiation protection behaviors when working in environments equipped with radiation protection protocols; however, most hospitals in Korea have not established specific radiation protection protocols for ER nurses [8]. There is a need to develop and implement guidelines, including those regarding the radioprotective equipment that needs to be worn by ER nurses, the safe distance from the radiation source to avoid harmful effects, the correct method for radiation shielding, and the response plan in the event of radiation exposure.

### 4.2. Strengths and Limitations

The objective of the present study was to identify factors affecting radiation protection behaviors by ER nurses, in order to help establish strategies to protect ER nurses from radiation exposure and promote health. However, because the study was limited to ER nurses in three regions, there are limitations in extending the results to a broader population. Although ER nurses are not legally designated as radiation workers at medical institutions, they are continually exposed to low-dose radiation due to the use of devices such as mobile X-ray machines. Unlike previous studies [8,15,18,19,20] that focused on radiation workers working in places with a large amount of radiation-emitting medical equipment, the present study only included ER nurses; the results are valuable because they specifically identify factors affecting radiation protection behaviors by ER nurses. These results can be used as basic data to support the establishment of policies to improve radiation protection environments for ER nurses in the future.

## 5. Conclusions

The factors affecting radiation protection behavior by ER nurses, in descending order, were wearing protective equipment, experience of radiation protection education, attitude towards radiation protection, and knowledge about radiation protection. The explanatory power of these factors for radiation protection behavior was 31.3%. In order to improve radiation protection behaviors by ER nurses, based on these results, it will be necessary to provide administrative support at the institutional level. This might include mandating radiation protection education and preparing radioprotective equipment and facilities. It will also be important to help nurses to improve radiation protection behaviors by developing expert knowledge of radiation protection and a positive attitude towards radiation protection. Radiation protection behaviors not only protect the individual’s health, but also help to improve the health of future generations and descendants, to enhance the well-being of the nation, and to reduce societal costs.

The following proposals are made based on the results of the present study. Institutional and administrative improvements are required at the level of organizations, in order to investigate the state of radiation protection facilities and protective equipment, and to improve the use of radioprotective equipment by ER nurses. In addition, further research is needed to investigate the various factors affecting radiation protection behaviors by ER nurses for different scales of hospital and different participant characteristics.

## Figures and Tables

**Table 1:** Radiation protection behaviors to the general characteristics of participants (*n* = 198).

Characteristics	Categories	*n* (%)	M ± SD	t/F	*p* (Scheffé)
Gender	Male	10 (5.1)	3.89 ± 0.87	1.74	0.083
	Female	188 (94.9)	3.43 ± 0.81		
Age (years)	20~29	85 (42.9)	3.40 ± 0.76	0.43	0.652
	30~39	56 (28.3)	3.41 ± 0.71		
	≥40	57 (28.8)	3.54 ± 0.96		
Spouse	Yes	90 (45.5)	3.46 ± 0.74	0.02	0.985
	No	108 (54.5)	3.45 ± 0.90		
Education level	College	54 (27.3)	3.44 ± 0.85	0.69	0.504
	Bachelor	135 (68.2)	3.42 ± 0.78		
	≥Master	9 (4.5)	3.77 ± 0.89		
Total career (years)	<2	36 (18.2)	3.67 ± 0.75	1.40	0.244
	2~4	49 (24.7)	3.41 ± 0.76		
	5~9	36 (18.2)	3.30 ± 0.72		
	≥10	77 (38.9)	3.42 ± 0.88		
Career in present unit (years)	<2	73 (36.9)	3.47 ± 0.85	0.28	0.758
	2~4	57 (28.8)	3.39 ± 0.80		
	≥5	68 (34.3)	3.45 ± 0.77		
Subjective health status	Bad	19 (9.6)	3.33 ± 0.85	1.28	0.280
	Average	125 (63.1)	3.39 ± 0.77		
	Good	54 (27.3)	3.59 ± 0.85		
Experience of radiation protection education	Yes	90 (45.5)	3.82 ± 0.73	6.32	<0.001
	No	108 (54.5)	3.15 ± 0.75		
Necessity on radiation protection education	Yes	190 (96.0)	3.48 ± 0.81	2.28	0.024
	No	8 (4.0)	2.82 ± 0.80		
Intention to participate in radiation protection education	Yes	177 (89.4)	3.45 ± 0.81	1.05	0.302
	No	21 (10.6)	3.28 ± 0.87		
Wearing of protective equipment	Yes	124 (62.6)	3.72 ± 0.75	6.56	<0.001
	No	74 (37.45)	3.01 ± 0.72		
Regular radiation-related health examinations	Yes	102 (51.5)	3.65 ± 0.76	3.60	<0.001
	No	96 (48.5)	3.25 ± 0.82		

**Table 2:** Degrees of knowledge about radiation protection, attitude towards radiation protection, and radiation protection behaviors (*n* = 198).

Variables	Mean ± SD	Min~Max	Range
Knowledge about radiation protection	10.56 ± 2.34	5.00~16.00	0~16
Attitude towards radiation protection	4.35 ± 0.53	1.85~5.00	1~5
Radiation protection behaviors	3.45 ± 0.81	1.00~5.00	1~5

**Table 3:** Correlations of variables (*n* = 198).

Variables	Knowledge about Radiation Protection	Attitude towards Radiation Protection	Radiation Protection Behaviors
	r (*p*)	r (*p*)	r (*p*)
Knowledge about radiation protection	1		
Attitude towards radiation protection	0.34 (<0.001)	1	
Radiation protection behaviors	0.37 (<0.001)	0.33 (<0.0001)	1

**Table 4:** Factors affecting radiation protection behaviors (*n* = 198).

Variable	B	SE	β	*t*	*p*
(Constant)	1.14	0.47		2.46	0.015
Knowledge about radiation protection	0.04	0.02	0.12	1.79	0.045
Attitude towards radiation protection	0.26	0.10	0.17	2.62	0.009
Experience of radiation protection education ^†^ (Yes)	0.43	0.11	0.27	4.06	<0.001
Wearing of protective equipment ^†^ (Yes)	0.48	0.11	.029	4.21	<0.001
Regular radiation-related health examinations ^†^ (Yes)	−0.04	0.11	−0.02	−0.36	0.720
Need for radiation protection education ^†^ (Yes)	0.26	0.25	0.06	1.06	0.292
R^2^ = 0.334, Adjusted R^2^ = 0.313, F = 15.99, *p* < 0.001					

^†^ Dummy variables; SE = standard error.

## Data Availability

The data presented in this study are available on request from the corresponding author.
